# Baseline status of the levels and distribution of rare, noble, and fissionable elements from the Northern Nile Delta black sand deposits

**DOI:** 10.1038/s41598-024-69453-w

**Published:** 2024-09-03

**Authors:** Rana E. Fakhry, Zekry F. Ghatas, Naglaa F. Soliman, Samir M. Nasr

**Affiliations:** 1 Department of Environmental Studies, Institute of Graduate Studies & Research, Alexandria University, Alexandria, Egypt; 2 Department of Marine Ecology, Faculty of Aquaculture and Marine Fisheries, Arish University, Arish, Egypt

**Keywords:** Economic heavy minerals, Distribution and sources, Black sands, Nile Delta, Environmental sciences, Environmental chemistry

## Abstract

This study examined the composition, distribution, and origins of rare, Noble, and fissionable elements for the first time in black sand deposits from the Northern Delta coastal region. The findings showed that among the elements under investigation, Fe, Ti, Mn, and Sn had the greatest mean levels, while Hf, Cd, and As had the lowest mean amounts. According to the study's elemental composition, black sand is thought to have economic worth for Ti, Zr, Hf, Sn, Ag, and W. The Zr, Co, Cd, Cu, Hf, V, W, and Zn correlation points to the same source origin. It is clear that the accessory mineral composition in the sediments under study especially the heavy ones controls the geochemical patterns of trace elements. The trace element concentrations of interest show a pattern of element variability related to the mineralogy of the sands, as indicated by the principal component analysis and cluster analysis. To explore and exploit heavy minerals in the research region, the study's findings are important.

## Introduction

Sand dunes and beach sand are examples of detritus sediments that build into economic heavy mineral deposits along the coast. The weathering of igneous and metamorphic rocks produces these deposits. Sediments are carried to coastal regions by rivers and Aeolian processes, where wind, tide, and water currents sort and concentrate them. Beach sand and sand dunes are the result of these processes, which cause layers of thick sediments to accumulate in a range of coastal depositional conditions^1^. The crystalline igneous and metamorphic rocks that were carried by the River Nile from South Sudan and the Ethiopian Plateau are represented by the deposits of black sand in Egypt^2^. Huge amounts of the six common commercial minerals—ilmenite, magnetite, garnet, zircon, rutile, and monazite—as well as other elements—such as uranium, thorium, zirconium, hafnium, titanium, and rare earth elements—that are required for the nuclear industry are contained in its deposits^3,4^.

These minerals have economic value and can be extracted and used in a variety of industries: zircon (ZrSiO_4_) is an important gemstone with multiple color forms that can be used in various forms of jewelry and can be used at nuclear fuel, where it is also an ore of the radioactive element thorium; magnetite (Fe_3_O_4_) is used as a toner in electrophotography; ilmenite (FeTiO_3_) is used at lightweight alloys that are used to manufacture a wide variety of high performance parts and tools, such as aircraft parts, artificial joints for humans, and sporting equipment like bicycle frames; garnet is useful for polishing glass, whereas rutile (TiO_2_) is used to color porcelain and glass as well as to make certain steel and copper alloys. The presence of thorium and less uranium in monazite makes it radioactive; other minerals found in black sand include cassiterite, chromite, apatite, enstatite, and small amounts of biotite, epidote, sturolite, sphene, tourmaline, and olivine and trace elements such as gold and Platinium Group Element (PEG)^5^. They also contain traces of gold, cassiterite, and minerals of other elements- Pb, Zn, Hg, Ag, Nb, and Pt^6^. They are extensively recognized in the beach areas of Rosetta, Damietta, North Sinai, and the coastal sand dunes of the El-Burullus-Baltim area^7^. They stretch from Abu Qir to the west and Rafah to the east (about 700 km length and ~ 20 m deep). A belt of coastal dunes has formed on the shore between Burg El Burullus settlement and Baltim resort. It stretches eastward after El Gharbiya drainage. It goes farther inland to meet the new international high way as its boundary^8^.

The distribution and concentration of heavy minerals throughout the Mediterranean Sea coast are primarily determined by the magnitude, specific gravity, and direction of the long shore current^9^. Numerous publications on mineralogy and economics have addressed these black sands^8,10–16^.

The type of occurrence, the extractability of specific elements (Zr, Hf, rare earth elements, Th, and even Ti) present in the mineral phases, and the exploitation of specific natural products of industrial interest like rutile, zircon, and ilmenite all influence the economic significance of the black sands^17^. Even though rare metals are widely available in Egypt, the quantity and economic value of these metals were not adequately reflected in the processing and beneficiation of the rare metal-bearing rocks. The need for rare metals has increased dramatically over the past few decades due to their wide range of industrial applications, particularly in the automotive and electronic industries^18^. Therefore, it is essential to locate rare elements resources in order to close the growing gap between supply and demand. Nevertheless, a thorough evaluation of the El-Burullus-Baltime black sands, particularly with regard to their geochemical properties, has not yet been carried out.

In this study, the aim is to establish a baseline of data and clarify the existing situation to facilitate future comparisons and correlations with comparable black sands in other locations. This includes an attempt to determine the distribution, levels, and provenance of the selected constituents. A new inventory will be made using the obtained results.

## Materials and methods

### Study area

**Figure 1:**
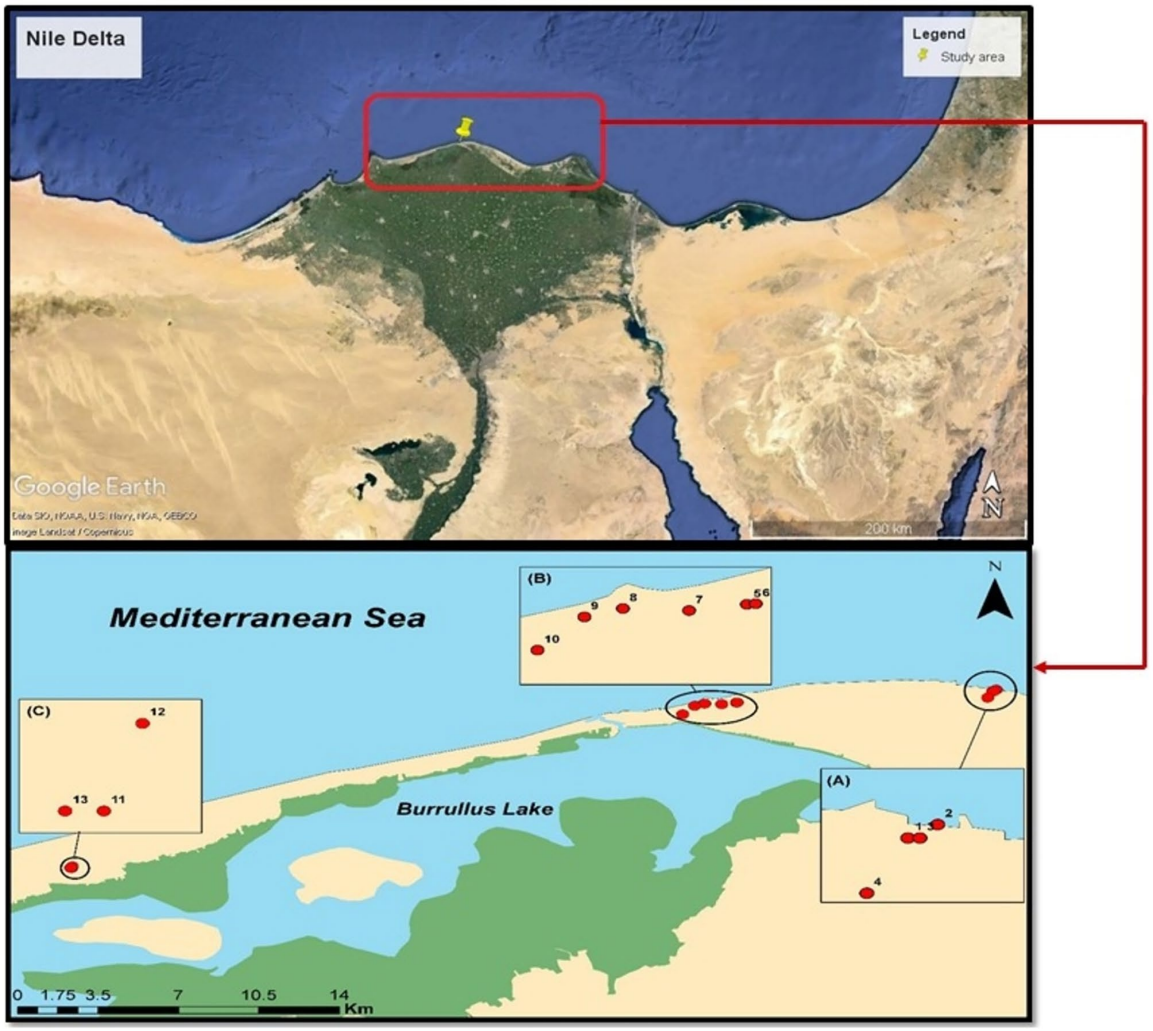
Sampling sites from the Northern Nile Delta black sand deposits (This figure was generated using ArcGIS 10.3.1; https://support.esri.com/en-us/patches-updates/2016/arcgis-10-3-1-for-desktop-engine-server-feature-service-2282 and Google Earth Pro; https://www.google.com/earth/about/versions/).

**Figure 2:**
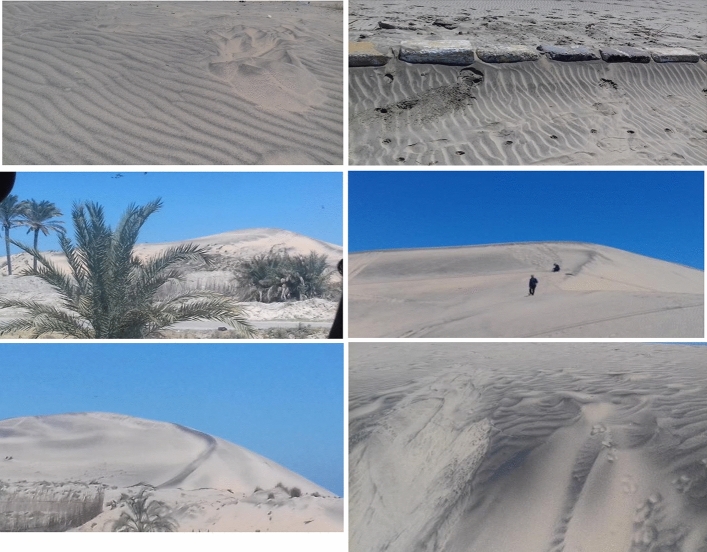
Field photos of the Northern Nile Delta black sand deposits.

The region between Burg EL-Burullus and Baltim, which includes beaches and coastal sand dunes, was the source of about 13 sedimentary samples. (Figs. 1 and 2). It is symbolized by belts of sand dunes and a level coastal plain^19^.

### Sample preparation and analysis

The original sand samples were dried and bromoform (Sp. Gr. 2.88 g/cm^3^) was used to prepare them for mineral separation^20^. Next, each separated sample is weighted to 0.5 g, and the EPA 3052A method is used to digest the samples in a closed system microwave (Anton Paar Multivalve PRO). To find (Sn, W, Hf, Zr, Ti, and Cr), the Alkaline Fusion Procedure was utilized^12^. ICP-OES (Agilent, Australia, Model: 5100 VDV) was used to determine the elements present in the samples. With its large depth field, high spatial resolution, and simple specimen preparation, scanning electron microscope with energy dispersive X-ray spectrometry (SEM/EDS) are an appropriate method most frequently utilized in soil, sediment and rock characterization. Scanning Electron Microscope (JSMIT200 In Touch Scope) supported by Energy-Dispersive X-Ray analyzing system (SEM–EDX) was applied in this study for measurements of Noble and fissionable elements.

### Quality control and quality assurance (QA/QC)

Spiked sample was done where a known quantity of analyte was added to a sample to test the accuracy of the method used. The recoveries were found to be higher than 90% for most of the investigated elements as follows: 110, 105, 75, 122, 110, 116, 106, 109, 110, 122, 118, 80, 119, 120, 122, and 82% for Ag, As, Cr, Co, Cd, Cu, Fe, Ti, Sr, Ni, Mn, Hf, V, W, Zn, and Zr, respectively. The manufacturer's handbook recommended settings were used for all instrumental settings. Ag, As, Cr, Co, Cd, Cu, Fe, Ti, Sr, Ni, Mn, Hf, V, W, Zn, and Zr had limits of detection (LODs) of 0.01, 0.004, 0.002, 0.0031, 0.001, 0.002, 0.1, 0.01, 0.001, 0.01, 0.01,0.01, 0.004, 0.04, 0.12, and 0.01 μg/g, in that order. All analyses were carried out in triplicate. For each run, three “blanks” were analyzed using the same procedure to check the purity of reagents and any possible contamination.

Scanning Electron Microscope equipped with the Energy-Dispersive X-Ray analyzing system (SEM–EDX) was used for characterization of Noble elements (Au), platinum group elements (Pt, Ir, and Os), and fissionable elements (Th and U). The mean value of three measurements at different locations was calculated. The standard soil sample (NCS DC 70333 CRM) was used for the quality control.

### Statistical analysis

A multivariate statistical method such as the principal component analysis (PCA) and cluster analysis (CA) is a dominant tool for assessing pollution levels among samples. The PCA method has been extensively applied in geochemical applications to recognize the origins of investigated elements. When combined with PCA, CA serves as a check for results and allows for the grouping of individual parameters and variable.

Pearson correlation analysis and principal component analysis (PCA) were applied using SPSS 20 software to the data to explore the correlations among the investigated elements. Furthermore, Hierarchical Clustering Dendrogram was used to group measured variables using MINITAB 17 software.

### Ethical approval

The current manuscript is not be submitted to another journal for simultaneous consideration or publish. All the methods included in the study are in accordance with the national guidelines.

## Results and discussion

### Heavy minerals content

Geologists' attention has recently been focused on Egypt’s black sand deposits because they offer a good source of heavy minerals rich in strategic and commercial metals, including zircon, garnet, rutile, ilmenite, magnetite, hematite, and monazite. The nuclear sector as well as other metallurgical and engineering industries require these economic minerals. Black sand deposits can also contain various placer minerals, including platinum, silver, gold, and cassiterite^15^.

**Figure 3:**
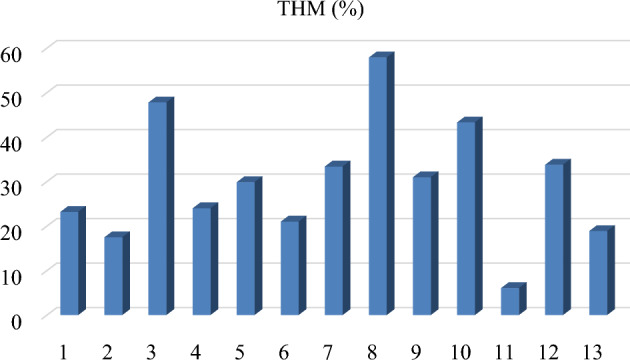
% of Total heavy minerals (THM) in the Northern Nile Delta black sand deposits.

The content of heavy minerals in the black sand deposits varied from 5.99 to 57.8% in this study (Fig. 3). The percentage of total heavy mineral concentration for some samples of placer sands from the Jhatipodar deposit in Odisha, India^21^, Astaranga beach in Puri, India^22^, the southeast portion of Korea's Yellow Sea coastline^23^, the Changjiang Yangtze River sediments, China^24^, and the Kinta valley in the State of Perak, Malaysia^25^, agreed with the average of 29.73% for all heavy minerals.

### Elements contents of black sand

#### Trace elements

Nanotechnology, ferro silicon alloys, and all smart sectors could benefit from the utilization of rare earth elements. In Egypt, trace elements are widely distributed and frequently coexist with other metals^26^.

Table 1 presents the results of the descriptive statistic of the element concentrations in the isolated heavy mineral fraction of the black sand samples. Hf, Cd, and As had the lowest mean contents among the elements examined, whereas Fe, Ti, Mn, and Sn had the greatest mean contents.

**Table 1:** Descriptive statistics for concentrations of elements in the investigated samples.

	Ag	As	Cr	Co	Cd	Cu	Fe	Ti	Sr	Ni	Mn	Hf	V	W	Zn	Zr	Sn
Min	1.6	1.7	500.9	71.7	7.5	15.5	36,867.4	31,555.7	157.3	46.9	1597.3	7.5	292.4	165.2	72.1	159.6	1172
Max	31.5	4.2	720	149.9	12.9	31.2	132,964.7	104,558.4	236.9	116.7	5190.9	17.1	470.8	228.4	119.9	490.1	1755
Average	12.59	3.30	611.62	112.47	10.02	24.48	106,665.96	76,792.95	196.36	58.92	2814.93	11.25	396.25	200.42	97.57	290.66	1428.38
SD	13.41	0.68	55.87	20.45	1.70	4.60	25,713.90	20,262.19	23.57	17.94	978.03	2.43	49.10	18.13	13.09	82.98	182.34
CV	106.50	20.63	9.13	18.19	16.98	18.78	24.11	26.39	12.01	30.44	34.74	21.65	12.39	9.05	13.41	28.55	12.77
Kurtosis	− 1.92	1.66	0.81	0.62	− 0.54	− 0.22	4.03	1.04	− 0.33	10.99	2.74	1.82	0.24	− 0.21	− 0.12	1.99	− 0.82
Skewness	0.58	− 1.14	0.099	− 0.13	0.17	− 0.29	− 1.82	− 1.21	− 0.06	3.20	1.74	0.88	− 0.59	− 0.46	− 0.26	0.76	0.26

With its unique magnetic properties and silvery white color, iron is a relatively dense metal. After oxygen, silicon, and aluminum, it is the fourth most prevalent element in the Earth's crust, making up 5% of its weight. The average concentration of iron in the research region is 106,666 µg/g, which is higher than the background Continental Crust of 56,300 µg/g^27^ and the upper continental Crust of 35,000 µg/g^28^. The Lake Nasser Nile sediments contain iron at concentrations ranging from 51,000 to 131,000 µg/g^29^ (Table 2) which correlates significantly with clay that due to terrestrial sources derived from weathering processes of igneous rock which interrelated to ilmenite and magnetite minerals.

**Table 2:** Comparison between average elements (μg/g) in the study area and other black sand deposits, background references, and sediment quality guidelines.

Location	Ag	As	Cr	Co	Cd	Cu	Fe	Ti	Sr	Ni	Mn	Hf	V	W	Zn	Zr	Sn
Study area	12.59	3.30	611.62	112.47	10.02	24.48	106,665.96	76,792.95	196.36	58.92	2814.93	11.25	396.25	200.42	97.57	290.66	1428.38
Northern Nile Delta^19^		2	343.2	44.5		28			292	59.5	883	140.5		32.6	43.5	4184.5	
Abu Khashaba^12^	309.32				33.56		185.894	435.06			596.45	3503.32	322.32		249.95	1077.26	
Sol Hamid^3^	31.06			234.14		28.64			44.50	96.99		1443.33			299.14		
El Quroun	100.24			297.98		14.32			37.20	297.03		4916.86			227.13		
Um Thagher^15^				170.72		11.01			28.40	180.26		1341.33			318.19		
Bir Shalateen^15^				262.88		13.57			16.60	109.85		1137.99			291.24		
Crustal average^27^	0.07	1.8	100	25	0.2	55	56,300	5700	375	75	950	3	135	1.5	70	165	2
Upper Continental crust^28^		1.5	83	17	0.098	25	35,000			44	600		107	2	71		5.5

In addition, the average concentration of manganese is 2814.9 µg/g which is higher than the average Continental Crust (950 µg/g)^27^, the upper continental crust (600 µg/g)^28^ and the Continental Crust (929 µg/g)^31^, that may be related to garnet and ilmenite minerals.

Titanium is a structural metal that is lightweight, strong, and resistant to corrosion. It is utilized in alloy form for high-speed aviation parts. It is predicted that the Earth’s crust contains 0.45 percent to 0.75 percent titanium in the form of TiO_2_^30^. The Nile River carries titanium that is carried there by weathering from igneous and sedimentary rocks. This study’s average titanium concentration, 76,793 µg/g, is higher than that of the average continental crust (5700 µg/g), and it is associated with the minerals ilmenite and rutile.

The mean hafnium content is 11.25 µg/g, surpassing both the average continental Crust’s (3 µg/g) and Upper Continental Crust's (5.8 µg/g) levels. This difference may be attributed to the zircon mineral. Arsenic, on the other hand, is produced by both natural and anthropogenic origins. The majority of crystalline rocks have low levels of it; basalt and granite have 1.5 µg/g and soil has 6 µg/g^32^. The current study’s average arsenic concentration of 3.3 µg/g is higher than the average continental crust's (1.8 µg/g)^27^ and the Upper Continental Crust (1.5 µg/g)^28^. The mineral arsenolite may be connected to this. Additionally, the mean cadmium concentration (Table 1) is 10.02 µg/g, greater than that of the average continental Crust (0.2 µg/g)^27^ and the upper continental crust (0.098 µg/g)^28^. This observation could potentially be linked to the ilmenite mineral.

Ag (106.50%), Mn (34.74%), and Ni (30.44%) have greater coefficients of variation (CVs) than the other elements. The high CV values showed that the elements' spatial distributions in the black sands were not uniform and that either human activity or distinct lithological units within the deposits might have an impact on their contents^33^. It is important to note that the changes in the concentration of elements found in these deposits of black sand are determined by and linked to changes in the contents of the heavy minerals found in these deposits.

The negative skewness values for As, Co, Cu, Fe, Ti, Sr, V, W, and Zn indicate that anthropogenic activities did not significantly affect the accumulation and regional variability of these metals. Table 1 shows that the skewness values of Ni and Mn were 3.2 and 1.7, respectively, whereas those of Cr, Cd, Hf, Sn, and Zr were less than 1. These findings showed that lithological variations or human activity in the region might have an impact on Mn and Ni^34^. Storm water runoff from the surroundings and the sewage input released into the Nile Delta ecosystem are the main routes of metals in the investigated sediments. The metals produced from industrial, sewage, irrigation, and urban runoff have accumulated over a long period of time in the surface sediment of the area.

**Figure 4:**
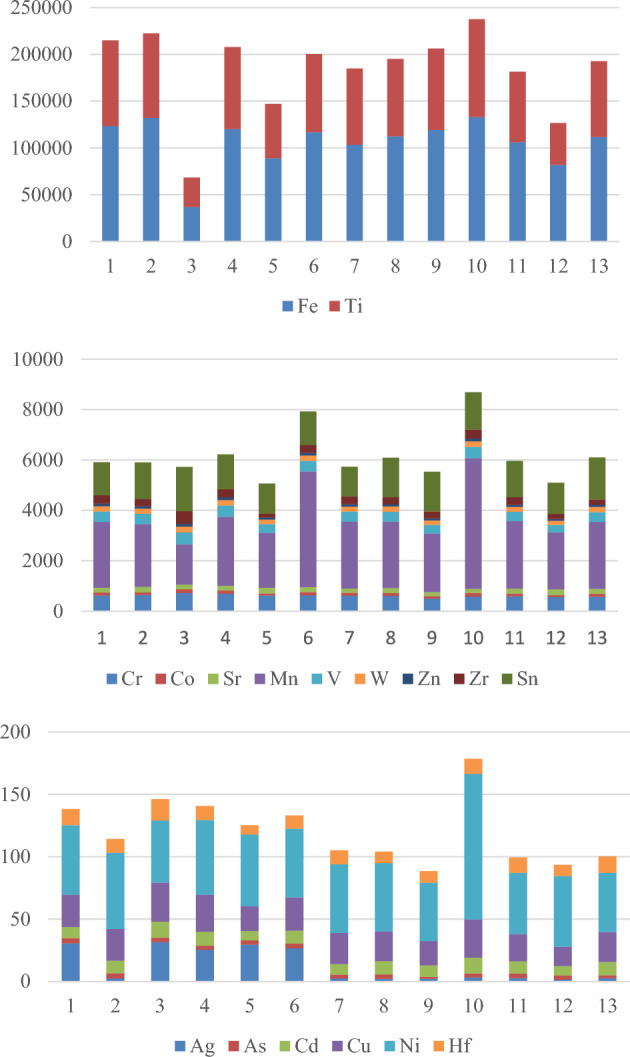
Concentrations of elements (ppm) in the investigated samples from the Northern Nile Delta black sand deposits.

It is evident from Fig. 4 that the investigated sediments display enrichment of trace elements such as Zr, Sr, Zn, and V. The concentration of zirconium varies greatly, from 159.6 to 490 μg/g. This is most likely because zircon crystals predominate.

Wolfenden^35^ and Garzanti^36^, and the pioneer conclusion of Shukri^37^ state that the fundamental volcanic rocks of the Blue Nile provenance are the source of these titanomagnetites minerals and elements V, Ni, Zn, and Zr. El-Kammar^38^ concurs suggesting that the high concentration of heavy metals (V, Zn, Ni, and Cu) primarily originates from the volcanic rocks.

Table 3 of the current study shows significantly greater concentrations of Mn, Co, Cr, W, and Zn than those found in the coastal sediments of the Northern Nile Delta^19^. Additionally, black sands from El Quroun, Um Thagher, and Bir Shalateen that were gathered from the southern Eastern Desert had greater Sr and Cu levels^15^. The levels of Co, Ni, Hf, and Zn were significantly lower than those found in the southern Eastern Desert locations of Sol Hamid, El Quroun, Um Thagher, and Bir Shalateen^15^.

**Table 3:** The content of PGE and feasible elements (%).

Site. No	Os	Ir	Pt	Au	Th	U
1	0.14 ± 0.23	0.45 ± 0.13	0.15 ± 0.13	0.25 ± 0.10	0.04 ± 0.33	0.82 ± 0.13
7	N.D	N.D	ND	ND	N.D	0.1 ± 0.04
8	0.85 ± 0.26	0.04 ± 0.17	0.53 ± 0.16	0.06 ± 0.14	N.D	0.38 ± 0.13
12	N.D	N.D	ND	ND	N.D	0.14 ± 0.09
13	0.20 ± 0.12	N.D	0.06 ± 0.08	ND	ND	0.13 ± 0.09
14	0.71 ± 0.18	0.26 ± 0.13	0.41 ± 0.12	0.35 ± 0.11	0.18 ± 0.09	0.08 ± 0.12

#### Noble and fissionable elements

Noble elements, such as Au and elements of the platinum group (Pt, Ir, and Os), are found in Egypt in a variety of locations, primarily in the Eastern Desert's basement complex^26^.

**Figure 5:**
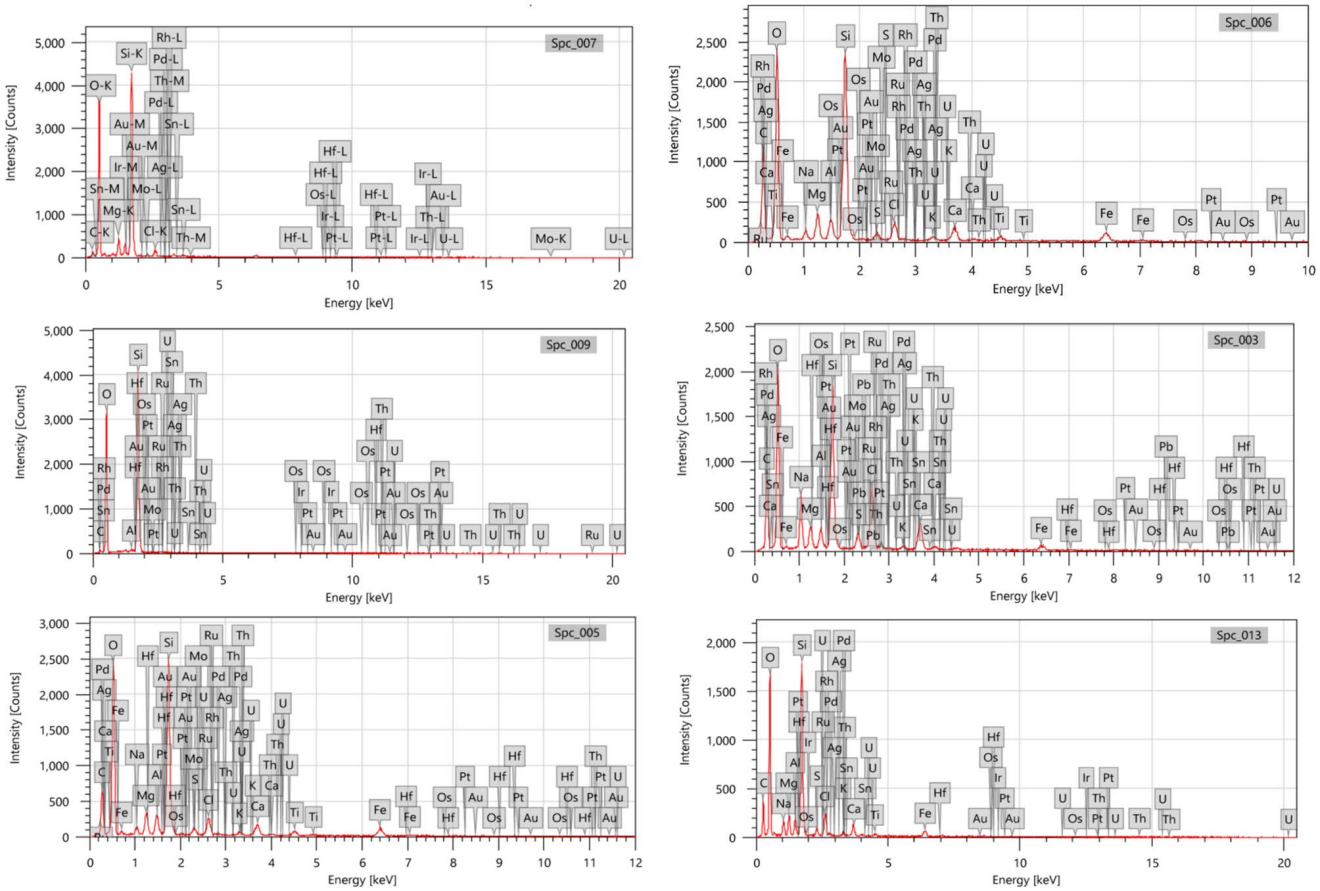
Scanning electron microscope SEM for samples 1, 7, 8, 12, 13, 14.

In this investigation, the average mass percentage (%) for the six raw samples analyzed using a scanning electron microscope (SEM) ranged from ND to 0.35 ± 0.011 for Au (Fig. 5). Because of the hot, dry climate of the Egyptian deserts, which caused physical weathering, the majority of placer gold deposits are found near auriferous quartz veins. Modern placers and lithified placers are the two main types of placer gold that have formed in Egypt^39^. Beach and alluvial placers are the two categories of recent placers.

Alluvial placer mining is favored by the significant concentration of mechanically scattered gold in the Eastern Desert wadis, according to Baioumy^26^. There have been trace amounts of gold found in beach placers on the Mediterranean Sea in some black beach sands in Egypt. Gold has recently been found in the locations of G. Nazar, G. Kamel, and Uwaynat. These locations have up to 14 g/t of gold^40^, which is significantly more than the study area’s (ND to 3.5 ± 0.11 g/t).

Mafic–ultramafic ophiolite rocks and certain layered intrusions or concentrically-zoned mafic ultramafics are typically linked to PGE^26^. The average mass percentage (%) of Pt ranged from ND to (0.53 ± 0.16), Ir from ND to (0.45 ± 0.13), and Os from ND to (0.85 ± 0.26), according to Table 3. These findings are significantly less than those found in the chromitites of Egypt’s southern, central, and eastern deserts, where Ahmed^41^ reports ND-70.8% for Os, ND to 53.6% for Ir, and ND to 47.4% for Pt.

In Egypt, fissionable elements such as U and Th can be found in a number of locations, mostly in the Eastern Desert^26^ basement complex. The average mass percentage (%) of thorium was not found at localities 7, 8, 12, and 13, and it was 0.18 ± 0.09 at station 14 and 0.04 ± 0.23 at localities 1, according to the results presented in Table 3 and Fig. 5. On the other hand, at localities 14 and 1, the average mass percentage (%) of uranium varied from 0.08 ± 0.12 to 0.82 ± 0.13. The presence of monazite and zircon with subordinate thorite, uranothorite, and xenotime may be connected to the increased Th and U contents.

One of the possible sources of Th in Egypt, according to Baioumy^26^, is monazite, which is made up of rare earth elements and Th phosphate ((Ce, La, Nd, Th)PO_4_) in the black sands along the Mediterranean Sea Coast. Abd El-Naby^42^ also discovered several thorium occurrences in granitic rocks in the Um Ara region of the south-eastern desert. The thorite grains have ThO_2_ values ranging from 68.4 to 72.1 weight percent, and UO_2_ contents that are greater than the amounts of Th and U found in this study, ranging from 5.9 to 7 weight percent.

### Statistical analysis

Using the normalized data, Pearson correlation coefficients were computed to determine the relationships between the various constituents in the El Burullus-Baltim sand dunes. The majority of the examined factors had positive correlations with one another, as Table 4 illustrates. Strong correlation of Mn with Fe (r = 0.56, *p* < *0.05*), Ti (r = 0.62, *p* < *0.05*) and Ni (r = 0.73, *p* < *0.01*) that relates to garnet and ilmenite minerals.

**Table 4:**
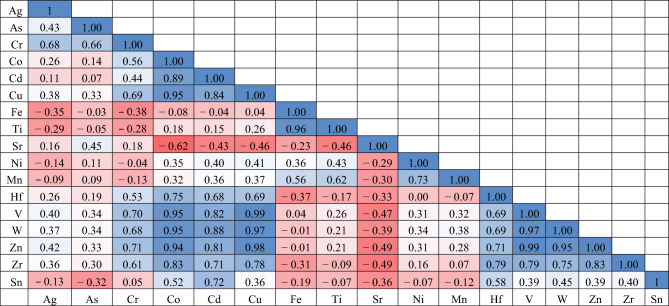
Person correlation coefficient between investigated elements.

**Figure 6:**
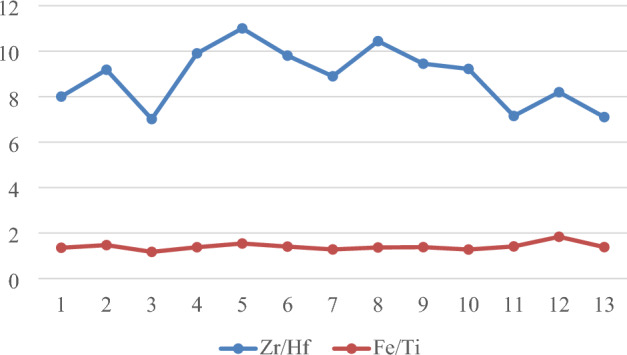
Zr/Hf and Fe/Ti ratios in the investigated sites from the Northern Nile Delta black sand deposits.

Fe/Ti ratio is usually determined for evaluation of titaniferous minerals (Martin & Long, 1960). Strongly ferromagnetic titanomagnetite samples are defined as those having high Fe/Ti ratios greater than 1. All samples’ Fe/Ti ratios are displayed in Fig. 6. In general, strong ferromagnetic titanomagnetite, a characteristic of heavy mineral sands, is present in all of the examined samples.

The weathering and breakdown of Ethiopian and Sudanese mountain ranges is thought to be the natural source of Fe, Mn, and Ti. Egypt’s heavy-mineral sands are concentrated by the sea44 and are a result of flooding in the higher stages of the Nile, which then travels down the river and its tributaries to the Mediterranean. Alluvial material that has been eroded from the higher parts of the river has naturally been sorted and separated by wind and rain, and the soluble minerals have dissolved.

Strong positive associations (*p* < *0.01*) between Cr and As and Ag were discovered. Cu and Cr, as well as Co and Cd, showed a significant positive connection (*p* < *0.01*). Zr and Co, Cd, Cu, Hf, V, W, and Zn (*p* < *0.01*) and Cr (*p* < *0.05*) showed a strong positive correlation, with the positive correlation between (Cu, Zn) and Co being related to the montmorillonite bound by chemisorption and exchange with Zn^2+^, Cu^2+^, and the strong positive correlation between Co and (Hf, W, V, Zr, Cr) possibly related to the bound with ilmenite mineral crystal lattice as traces element^43^.

Furthermore, there is a significant positive association (r = 0.79) between Hf and Zr. That might be connected to the mineral zircon, ZrSiO_4_. This crystallization is derived from metamorphic, sedimentary and volcanic rocks. The element HfO_2_, which is chemically related to hafnium and has a concentration in zirconia minerals ranging from 0.5 to 2.0% by weight, can be formed into the crystal lattice of zircon. Because zircon's structure contains Th and U, it can be regarded as radioactive^44^.

Moreover, the ration between Zr and Hf are frequently used in sediment source discrimination^45^. The Zr/Hf ratio is less than the chondritic value of approximately^30^, ranging from 15.05 to 29.53 (average: 25.79) (Fig. 6). Moreover, during simple magmatic differentiation^46^, the Zr/Hf ratio typically declines from ultramafic to felsic, indicating that the zircon under study may have originated from mafic rocks.

Strong positive correlation (*p* < *0.01*) has been found between vanadium and (Cr, Co, Cd, Cu, Hf, W, Zn, and Zr). This suggests a possible relationship between vanadium and naturally occurring source rocks, such as metamorphic and igneous rocks that are delivered through the Nile River and weather to become ilmenite, garnet, and rutile. Sr does not, however, correlate with any other element, with the exception of Co, which has a negative correlation (r = − 0.62; *p* < *0.05*) with Sr, suggesting their incorporation in discrete minerals or their separate mobilization.

Tungsten is strongly positively correlated with Cr, Co, Cd, Cu, Hf, V, Zn, and Zr), it may be related to natural source rocks as metamorphic and igneous rocks which delivered by weathering through river Nile^47^. Principal component analysis (PCA) is a tool that may be used to investigate potential sources of heavy metals from both natural and anthropogenic sources, as well as to evaluate the relationship between the elements under investigation^48^.

**Figure 7:**
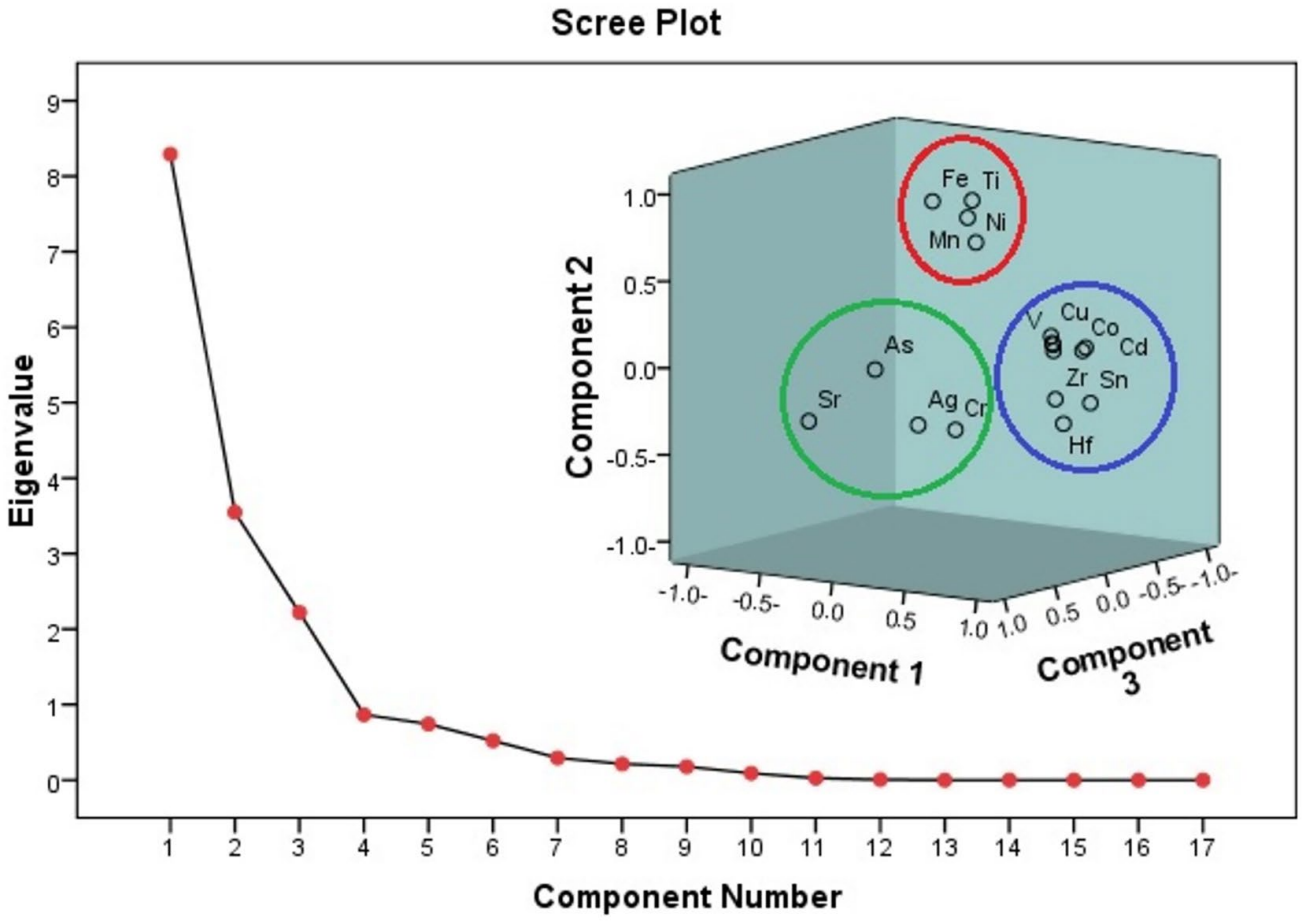
PCA of elements by scree plot of the characteristic roots (Eigen values), and component plot in rotated space.

Principal component analysis (PCA) with varimax rotation was performed to categorize the possible sources of investigated elements. Three principal components (PCs) with eigenvalues > 1 were found, explaining 82.702% of all variability (Fig. 7). The PC1 with 48.776%, contribution, Co, Cd, Cu, Hf, V, W, Zn and Zr had loadings of 0.973, 0.887, 0.980, 0.776, 0.979, 0.971, 0.979, and 0.861, respectively (Fig. 7 and Table 5). Co made strong positive correlations with Cd, Cu, Hf, V, W, Zn and Zr (r = 0.887, 0.947, 0.746, 0.946, 0.949, 0.940, and 0.828, respectively) and Cd had strong correlations with Cu, Hf, V, W, Zn, and Zr (r = 0.841, 0.683, 0.822, 0.881, 0.809, and 0.705, respectively).

**Table 5:** Factor loadings on elements in black sand deposits of the Nile Delta (n = 13).

Element	PC1	PC2	PC3
Ag	0.377	− 0.535	0.429
As	0.302	− 0.313	0.773
Cr	0.672	− 0.563	0.407
Co	0.973	0.077	− 0.141
Cd	0.887	0.128	− 0.236
Cu	0.980	0.079	0.123
Fe	− 0.071	0.855	0.285
Ti	0.159	0.883	0.197
Sr	− 0.486	− 0.508	0.477
Ni	0.344	0.606	0.266
Mn	0.309	0.732	0.337
Hf	0.776	− 0.305	− 0.294
V	0.979	0.045	0.101
W	0.971	0.039	0.085
Zn	0.979	0.005	0.080
Zr	0.861	− 0.217	− 0.118
Sn	0.480	− 0.036	− 0.726
Eigenvalue	8.29	3.55	2.22
% variance explained	48.776	20.865	13.061
Cumulative % variance	48.776	69.641	82.702

Extraction method: Principal component analysis.

Rotation method: Varimax with Kaiser Normalization.

Similar to W, Zn, and Zr, V had substantial correlations with them (r = 0.969, 0.9963, and 0.794, respectively), indicating that these elements might come from the same source. While there is a substantial positive connection between Co and (Hf, W, V, Zr, Cr), this correlation may be attributed to the association with the ilmenite mineral crystal lattice as traces element^43^. The high connections between Zn contents and Co, Cr, and Zr, as shown by Moufti and Arafa^49^, are undoubtedly influenced by lithogenic components present in the original source.

The elements Fe, Ni, Ti, and Mn are grouped together in PC2 of 20.865%. PC2 describes natural sources that sediment buildup and parent rocks are thought to be responsible for. Iron has a strong association with Ti and Mn, which are related to the minerals magnetite and ilmenite, respectively. Magnetite is a common iron oxide (Fe_3_O_4_) mineral that can be found in sedimentary, metamorphic, and igneous rocks. However, there is a significant link between Mn and Ti and Ni (r = 0.620 and 0.732, respectively), which is related to the minerals ilmenite and rutile.

Ag, As, Sr, and Cr clustered together with loadings of 0.429, 0.773, 0.477, and 0.407, respectively, and PC3 contributed 13.06%. Ag and Cr have a strong positive association (r = 0.678), which could be explained by the rutile mineral’s natural source^44^. Furthermore, As is used a pathfinder element for silver and other precious metals, as shown by numerous published works^50^.

**Figure 8:**
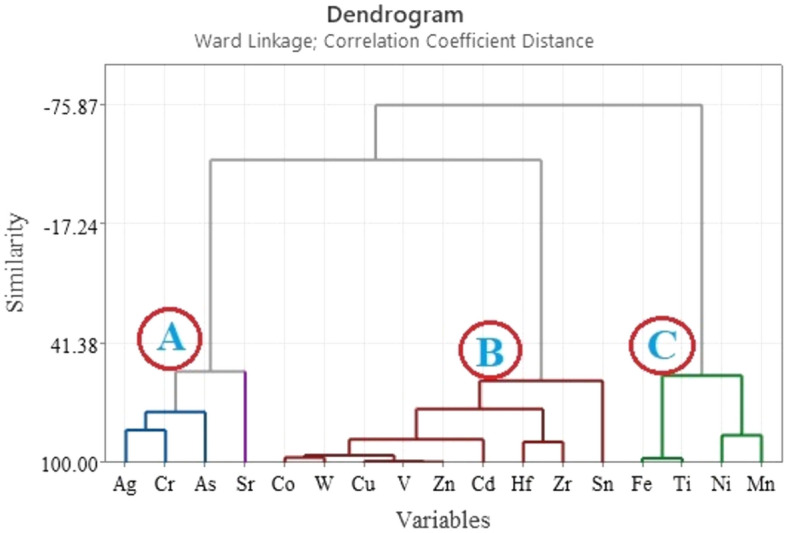
Clustering of elements in separated heavy mineral fraction of the investigated black sand deposits.

Cluster analysis is a multivariate statistical technique that is frequently used in environmental research to find groups or clusters of same variables based on similarities^51^. This approach is thought to be highly effective and produces clusters that are comparatively stable and well-organized^52^. The element data set was used in the current work’s cluster analysis, which was then displayed in a two-dimensional dendrogram plot as seen in Fig. 8. There are three clusters in this dendrogram. Cluster I includes Ag, Sr, Cr, and As. The great degree of similarity among the elements in this initial cluster group suggests that the rutile mineral may be the primary source of these elements.

The components in Cluster II—Co, W, Cu, V, Zn, Cd, Hf, Zr, and Sn—indicate that the parent rock in the research area is where these elements originate from weathering. Mn, Fe, Ni, and Ti make up Cluster III. These elements have a high degree of similarity within this cluster group, suggesting that their primary source may be natural minerals such as ilmenite and garnet. One important source of titanium is thought to be ilmenite (Fe, Mg, Mn) TiO_3_ which is found in the black sand deposits of Egypt^53^. Both Pearson correlation analysis and PCA concur well with these results.

### Processing and extraction of rare metals in Egypt

Despite the fact that rare metals are widely distributed in Egypt, very few efforts have been made to process and extract them, in relation to their potential for occurrence and economic significance^26^.

Regarding the commercial scope of rare metals extraction, the only endeavors that have been successful in Egypt to date have involved the prospecting and extraction of gold from many mines located in the Eastern Desert's Sukkary (Centamine Limited) and Hamash (Hamash Co.) regions. Commercial production employing surface, open pit, open cast mining techniques has just recently begun in both regions^54^.

Along the coast of the Mediterranean Sea are extensive reserves of black sand. As a result, in addition to using these black sands for the potential extraction of Th and rare earth elements, they can also be used to extract minerals that are considered as potential sources of metals like zinc from zircon, iron from hematite and magnetite, and titanium from rutile and ilmenite. These metals have a wide range of industrial uses, including steel and its alloys (Fe), the production of a diverse range of metal parts that require extremely high strength and low weight, such as artificial joints for humans, aircraft parts, and sporting goods like bicycle frames made of Ti, and a variety of high temperature uses for Zr. Therefore, the combined uses of these metals along with the rare metals can significantly increase the value of Egypt’s black sands deposits. In some cases, black sand deposits contain additional placer minerals such as gold, silver, platinum, and cassiterite^15^. Monazite, zircon and rutile minerals do contain a number of elements necessary for the nuclear industry e.g. uranium, thorium, zirconium, hafnium, titanium and rare earths elements^55^.

In general, Egyptian black sand contains a variety of economically valuable minerals. It is a possible source of rare earth elements, specifically Th and U. Rare earth elements could be used in all smart sectors, including nanotechnology and ferro silicon alloys. As a result, the Egyptian black sands contain considerable quantities of metal-rich heavy minerals with strategic importance and commercial value^56^. In this study, black sands have reasonable concentrations of the following elements: titanium (Ti), vanadium (V), chromium (Cr), manganese (Mn), iron (Fe), zinc (Zn), arsenic (As), zirconium (Zr), cadmium (Cd), hafnium (Hf), silver (Ag), tungsten (W), and tin (Sn) as compared to the background crustal average (Table 2). The enrichment of these elements relative to the background crustal average indicated that the black sands could be a source of co-production of these elements.

Because of their radioactive mineral composition (e.g., zircon, uranothorite, and comparatively monazite), the Egyptian black sands in the north are the focus of significant studies by the Nuclear Materials Authority of Egypt for the extraction of uranium that is required to form a “yellow cake”.

Such material emphasizes that Egyptian black sands are prospective sources of radionuclides, rare-earth elements, Fe, and Ti, making them worthy of international and local tenders in the context of the state’s new mining and investment regulations, which were formulated in 2016–2017^57^.

## Conclusion

The Egyptian black sand deposits are found as coastal sand dunes and beach sediments, and they contain enormous stocks of heavy minerals rich in valuable metals. The baseline status of As, Cr, Co, Cd, Cu, Fe, Ti, Sr, Ni, Mn, Hf, V, W, Zn, Zr, and Sn in the separated heavy minerals fraction from the Northern Nile Delta black sand deposits that could be economically exploited is documented in this work. Additionally, it sheds light on the origins of these elements and identifies possible sources using multivariate statistical techniques. In order of abundance, the elements were Fe > Ti > Mn > Sn > Cr > V > Zr > W > Sr > Co > Zn > Ni > Cu > Ag > Hf > Cd > As. Nonetheless, the study's statistical analysis suggests that the placer deposits of black sand in the study region may have commercial value for Ti, Zr, Hf, Sn, Ag, and W. The trace elements content of the heavy minerals fraction of the investigated Northern Nile Delta black sand placer deposits reflects the nature of its mineral composition and their source rocks.

This work provides a solid foundation for future investigations on the composition, distribution and sources of rare, Noble and fissionable elements in black sand deposits of the Northern Delta coastal region. Thus, the integrated utilizations of these economic elements can add significant values to the black sands deposits in Egypt.

## Data Availability

The data that support the findings of this study are available upon request from the corresponding author.
